# Carbonic Anhydrase III Promotes Cell Migration and Epithelial–Mesenchymal Transition in Oral Squamous Cell Carcinoma

**DOI:** 10.3390/cells9030704

**Published:** 2020-03-13

**Authors:** Yin-Hung Chu, Chun-Wen Su, Yih-Shou Hsieh, Pei-Ni Chen, Chiao-Wen Lin, Shun-Fa Yang

**Affiliations:** 1 Institute of Medicine, Chung Shan Medical University, Taichung 402, Taiwan; spitwater9@hotmail.com; 2 Department of Medical Research, Chung Shan Medical University Hospital, Taichung 402, Taiwan; jeff11041986@gmail.com; 3 Institute of Biochemistry, Microbiology and Immunology, Chung Shan Medical University, Taichung 402, Taiwan; csmcysh@csmu.edu.tw (Y.-S.H.); peini@csmu.edu.tw (P.-N.C.); 4 Institute of Oral Sciences, Chung Shan Medical University, Taichung 402, Taiwan; cwlin@csmu.edu.tw; 5 Department of Dentistry, Chung Shan Medical University Hospital, Taichung 402, Taiwan

**Keywords:** CA III, epithelial–mesenchymal transition, oral cancer, metastasis

## Abstract

Epithelial–mesenchymal transition (EMT) is strongly correlated with tumor metastasis and contains several protein markers, such as E-cadherin. Carbonic anhydrase III (CA III) exhibits low carbon dioxide hydratase activity in cancer. However, the detailed mechanisms of CA III and their roles in oral cancer are still unknown. This study established a CA III-overexpressed stable clone and observed the expression of CA III protein in human SCC-9 and SAS oral cancer cell lines. The migration and invasion abilities were determined using a Boyden chamber assay. Our results showed that the overexpression of CA III protein significantly increased the migration and invasion abilities in oral cancer cells. Moreover, a whole genome array analysis revealed that CA III regulated epithelial–mesenchymal transition by reducing the expression of epithelial markers. Data from the GEO database also demonstrated that CA III mRNA is negatively correlated with CDH1 mRNA. Mechanistically, CA III increased the cell motility of oral cancer cells through the FAK/Src signaling pathway. In conclusion, this suggests that CA III promotes EMT and cell migration and is potentially related to the FAK/Src signaling pathway in oral cancer.

## 1. Introduction

Oral cancer has become a common cancer among humans, with more than 90% of cases being oral squamous cell carcinoma [1,2]. Tumor metastasis has been a major problem in the clinical treatment of various types of cancer [3,4,5]. Epithelial–mesenchymal transition (EMT) is a process in which epithelial cells transform into mesenchymal cells. Several characteristics of change accompany the process of EMT changing from a polygonal to spindle shape; for example, apico–basolateral polarization turns into anterior–posterior polarization, and strong cell-to-cell adhesion becomes focal cell-to-cell contact and also increases the cell migration potential [6,7,8]. The EMT process includes the downregulation of epithelial markers E-cadherin, claudin, and cytokeratins, as well as the upregulation of mesenchymal makers N-cadherin, vimentin, and fibronectin [9,10]. Relevant studies have indicated that EMT-related molecules are connected to invasion and metastasis in oral cancer [11,12,13,14,15] and that the loss of E-cadherin is also associated with the EMT process, which causes tumor metastasis [16,17].

Carbonic anhydrase III (CA) is a family of metalloenzymes, and its active site contains a zinc ion [18]. The main function of CA is to catalyze carbon dioxide into bicarbonate as a reversible hydrolysis reaction. CA participates in carbon dioxide transport, calcification, and photosynthesis. In mammalian physiological functions, CA regulates ion transport, the pH value, and water homeostasis, and takes part in the synthesis of glycogen, urea, and lipid during metabolism [19,20,21,22]. CA III is located on chromosome 8q22 and has a strong ability to hydrolyze carbon dioxide [23]. CA III demonstrates obvious expression in skeletal muscle, which helps carbon dioxide to move to tissue capillaries. High levels of CA III expression have also been discovered in the spleen, kidney, lung, and heart [24].

In recent years, CA inhibitors, including acetazomide, methazolamide, ethoxzolamide, dichlorophenamide, dorzolamide, and brinzolamide, have been used in laboratory cancer studies. Some studies have suggested that CA inhibitors could significantly reduce cancer cell growth, cell proliferation, migration, and colony formation, both in vivo and in vitro [25,26,27,28]. Moreover, Dai et al., demonstrated that CA III promotes the cell invasion capability in hepatocellular carcinoma cells through the FAK signaling pathway [29]. However, insufficient evidence supports the relation of CA III to oral cancer or tumor metastasis. Therefore, this study established a CA III overexpression system to clarify the roles of CA III in oral cancer development and metastasis.

## 2. Materials and Methods

### 2.1. Cell Culture

Human oral squamous cell carcinoma (OSCC) cell lines SAS and SCC-9 were purchased from the Japanese Collection of Research Bioresources Cell Bank (JCRB, Shinjuku, Japan) and were cultured in DMEM/F-12 medium (Life Technologies, Grand Island, NY, USA) with 10% fetal bovine serum (FBS). All cell lines were maintained at 37 °C in a humidified atmosphere of 5% CO2. 

### 2.2. CA III Overexpressed System

SAS and SCC-9 cell lines were used for the target cell lines to establish the stable CA III overexpressed cell clones. The pEGFPN-1 vector (Promega Corp., Madison, MI, USA) was chosen due to it being easier to analyse the transfection efficiency by a fluorescence microscope. The forward primer 5′-cacgaattcATGGCCCAAGGAGTGGGGC-3′ and reverse primer 5′-gtgggatccctTTTGAAGGAAGCTCTCACCA-3′ containing the EcoRI and BamHI restriction sites, respectively, were used to amplify the CA III sequence. The products, after having been treated with EcoRI and BamHI restriction enzymes, used ligase reagent to complete the ligation with the pEGFPN-1 vector. Then, 4 × 10^5^ cells were spread in a 6 cm culture dish that was incubated for 16 h and Lipofectamine 2000 reagent (Invitrogen, Carlsbad, CA, USA) was used to transfect the vector plasmid. After 16 h incubation, we used the G418 antibiotic to select stable clones and employed them as the target cells in the following experiments. The empty vector GFP was used as the control group compared to the GFP-CA III group.

### 2.3. RNA Interference Experiments

The human small interfering ribonucleic acids (siRNA) for CA III and scrambled siRNA were obtained from Ambion Inc. The CA III sense siRNA sequence was GCCGAGUUGUAUUUGAUGAtt and the CA III antisense siRNA sequence was UCAUCAAAUACAACUCGGCag. Cells were transfected with siRNA using Lipofectamine RNAiMAX reagent (Invitrogen, Carlsbad, CA, USA).

### 2.4. Migration and Invasion Assay

For the wound healing assay, cells with pEGFPN-1 CA III overexpressing vectors were plated in 6-well plates for 16 h, wounded by scratching with a pipette tip, and then incubated with DMEM/F12 medium containing 0.5% FBS for 12 or 24 h. Cells were photographed using a phase-contrast microscope. The cell migration ability was briefly estimated by measuring the wound recovered area. Additionally, the Boyden chamber (Neuro Probe, Cabin John, MD, USA) was changed for the migration and invasion assay. For the migration assay, cells were harvested and seeded to the chamber in serum-free medium and then incubated for 24 h at 37 °C. The invasion assay was carried out as described in the migration assay with a coating of Matrigel [30].

### 2.5. Reverse-Transcription PCR and Real-Time PCR

Total RNA was isolated from cultured cells using the Geneaid Total RNA Mini Kit (Geneaid Biotech Ltd., Taiwan), according to the manufacturer’s instructions. For reverse transcription, 2 μg of RNA was reverse-transcribed into cDNA using the SuperScript III First-Strand Synthesis Supermix kit (Invitrogen, Carlsbad, CA, USA). The mRNA levels of CA III, E-cadherin, vimentin, Slug, Twist, and GAPDH were examined through RT-PCR and real-time PCR, as previously described [31]. 

### 2.6. Western Blot

For Western blot analysis, equivalent amounts of total protein of cell extracts were used on 10% sodium dodecyl sulfate-polyacrylamide gel electrophoresis (SDS-PAGE) and overnight with antibodies CA III, E-cadherin, vimentin, Slug, Twist, total-Src, p-Src, total-FAK, p-FAK (Y397), and β-actin. Protein expression was detected by a chemiluminescence commercial kit (Amersham Biosciences, Buckinghamshire, UK). The relative photographic density was quantitated by scanning the photographic negatives on a gel documentation and analysis system (Alpha Innotech Corp., San Leandro, CA, USA) [32].

### 2.7. Gene Expression Microarray

The total RNA was commission Phalanx Biotech Group work with a whole genome array. Each sample needs 6 μg of RNA and the OD260/OD280 ≥ 1.8; OD260/OD230 ≥ 1.5 as the standard RNA quality. The Human OneArray Gene Expression Microarray kit was used to quantify the gene expression and analyse the data with a chart. The selection of EMT-related genes for the heat map was conducted according to the Human EMT RT^2^ Profiler PCR Array (QIAGEN), and the heat map was produced by HemI.

### 2.8. Luciferase-Report Assay

SCC-9 and SAS cells were spread 4 × 10^4^ cells per well in 24-well culture plates. After being incubated for 16 h, pGL3-basic, pGL3-control, and pGL3-E-cadherin promoter plasmids were co-transfected with the β-galactosidase expression vector (pCH110) into target cells by Turbofect (Fermentas, Carlsbad, CA, USA), as previously described [33]. After transfection for 24 h, the cell lysates were harvested, and the luciferase activity was determined by a luciferase assay kit. The values of the luciferase activity were normalized to the transfection efficiency and monitored by β-galactosidase expression.

### 2.9. Statistical Analyses

Statistics were calculated using student’s t-test (Sigmastat, Jandel Scientific, and San Rafael, CA, USA) to compare each group. Statistical significance was set at *p* < 0.05, and the values presented are the means ± standard deviation and were determined by at least three independent experiments.

## 3. Results

### 3.1. Effect of CA III on Cell Growth, Motility, Migration, and Invasion in oral Cancer Cells

**Figure 1:**
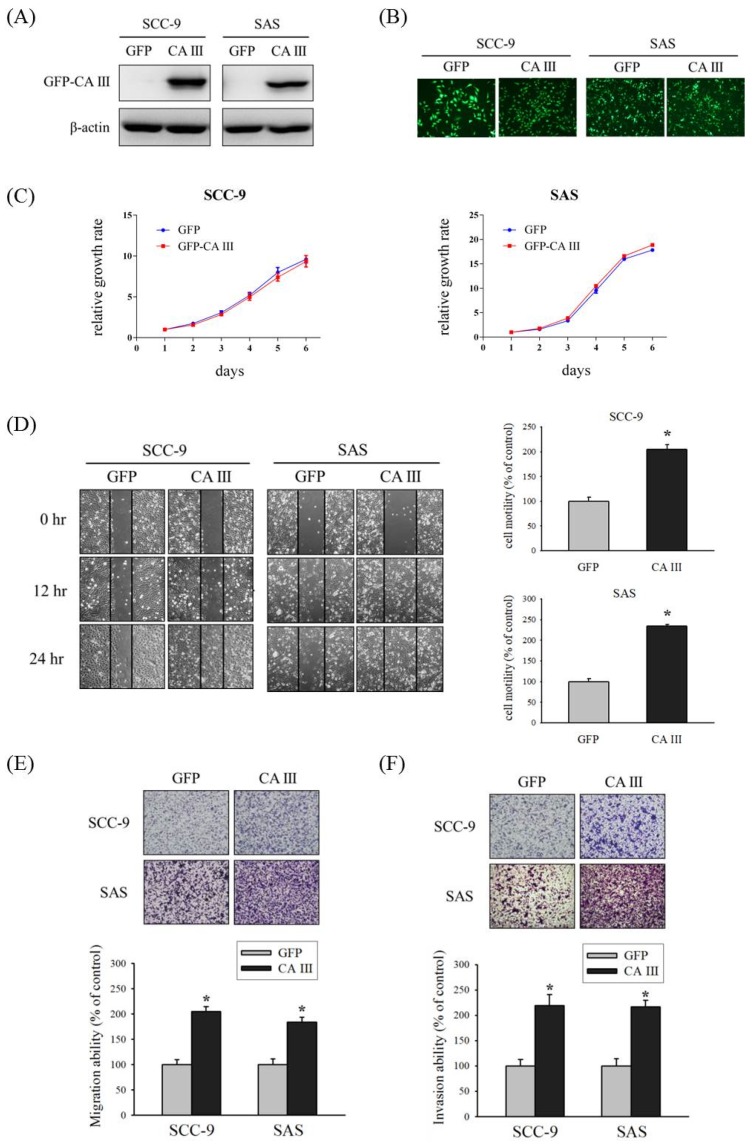
Effect of carbonic anhydrase III (CA III) on cell growth, motility, migration, and invasion in oral cancer cells. (**A**) Western blot of SCC-9 and SAS CA III stable clones, where β-actin was used as the internal control. (**B**) GFP and GFP-CA III expression were observed by fluorescence microscopy. (**C**) Growth curves of SCC-9 and SAS were analyzed by the MTT assay after the transfection of GFP or the GFP-CA III vector for 48 h. (**D**) SCC-9 and SAS CA III stable clones were wounded for 0, 12, and 24 h. Phase-contrast pictures of the wounds at three different locations were taken. (**E**) Migration ability of SCC-9 and SAS CA III stable clones were measured after 24 h. (**F**) Invasion ability of SCC-9 and SAS CA III stable clones were measured after 48 h. * *p* < 0.05 compared with GFP.

First, we established GFP-control and GFP-CA III stable cells of SCC-9 and SAS oral cancer cell lines, and checked the CA III protein expression and GFP expression by Western blot (Figure 1A) and fluorescence microscopy (Figure 1B). Next, we observed the effect of CA III on cell growth by the overexpression of CA III. The results suggested that CA III overexpression did not affect cell growth in both SCC-9 and SAS cell lines (Figure 1C). To determine the role of CA III in oral cancer cells, we used a wound healing assay to observe the cell motility by recovering the wound. The CA III overexpression group had a substantially greater wound area recovery ability compared with the GFP control group in both SCC-9 and SAS CA III stable cell lines (Figure 1D). Because CA III overexpression affected cell motility, we considered its cell migration and invasion ability to be similar to tumor metastasis behavior. Therefore, we used a Boyden chamber assay to analyze the cell migration and invasion abilities in a CA III overexpression system. The outcomes revealed that the weather migration (Figure 1E) or invasion (Figure 1F) ability was significantly increased in the CA III overexpression group.

### 3.2. CA III Regulates EMT Markers in Oral Cancer Cells

**Figure 2:**
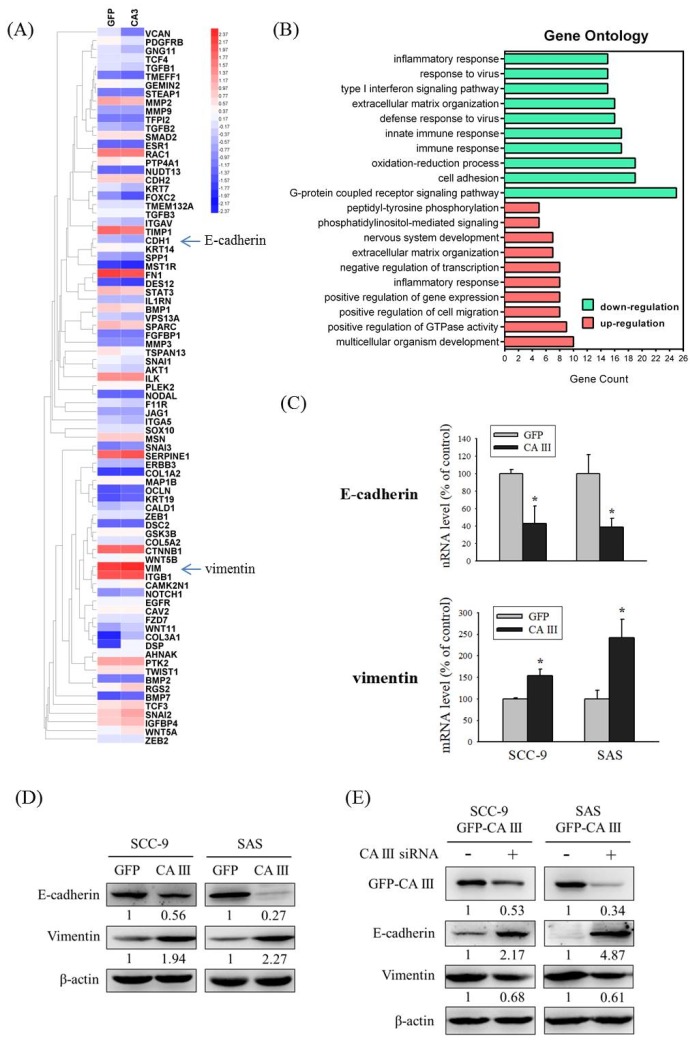
CA III regulates epithelial–mesenchymal transition (EMT) markers in oral cancer cells. (**A**) Heat map including 84 EMT-related genes in SCC-9 GFP and SCC-9 CA III cells was assessed by Human OneArray^®^. Blue arrows indicate the downregulation of E-cadherin (CDH1) and upregulation of vimentin (VIM) in SCC9 CA III cells. (**B**) Gene Ontology analysis for up-regulation and down-regulation genes between SCC-9 GFP and SCC-9 CA III cells was analyzed by a functional annotation tool (DAVID Bioinformatics Resources 6.8). (**C**) The mRNA levels of EMT markers E-cadherin and vimentin were analyzed by real-time PCR. The relative mRNA expression was normalized to GAPDH. * *p* < 0.05 compared with the GFP. (**D**) The protein expressions of EMT markers E-cadherin and vimentin were analyzed by Western blot in GFP and CA III stable cells. β-actin was used as the loading control. (**E**) The protein expression of EMT markers E-cadherin and vimentin after transfection with scrambled siRNA or CA III siRNA in CA III stable cells. β-actin was used as the loading control.

CA III overexpression, which induces cell migration and invasion abilities, may relate to several mechanisms. To clarify these mechanisms, we selected SCC-9-GFP-CA III overexpression stable clones and contrasted the mRNA changes under the CA III overexpression system by an mRNA array. The chart revealed that E-cadherin (CDH1) and vimentin (VIM) exhibited obvious expression differences that were related to EMT (Figure 2A). In addition, Gene Ontology analysis for up-regulation and down-regulation genes between SCC-9 GFP and SCC-9 CA III cells was analyzed by a functional annotation tool (DAVID Bioinformatics Resources 6.8) (Figure 2B). We also used a real-time PCR assay and Western blot assay to detect changes in E-cadherin and vimentin in the CA III overexpression system. The results suggested that CA III overexpression significantly decreased E-cadherin expression and increased vimentin expression at both the mRNA and protein level (Figure 2C and D). Moreover, the protein expressions of E-cadherin and vimentin were reversed after CA III knockdown by CA III siRNA transfection (Figure 2E).

### 3.3. CA III Inhibits the Promoter Activity of E-Cadherin and Promotes EMT-Related Transcription Factors Slug and Twist in Oral Cancer Cells

**Figure 3:**
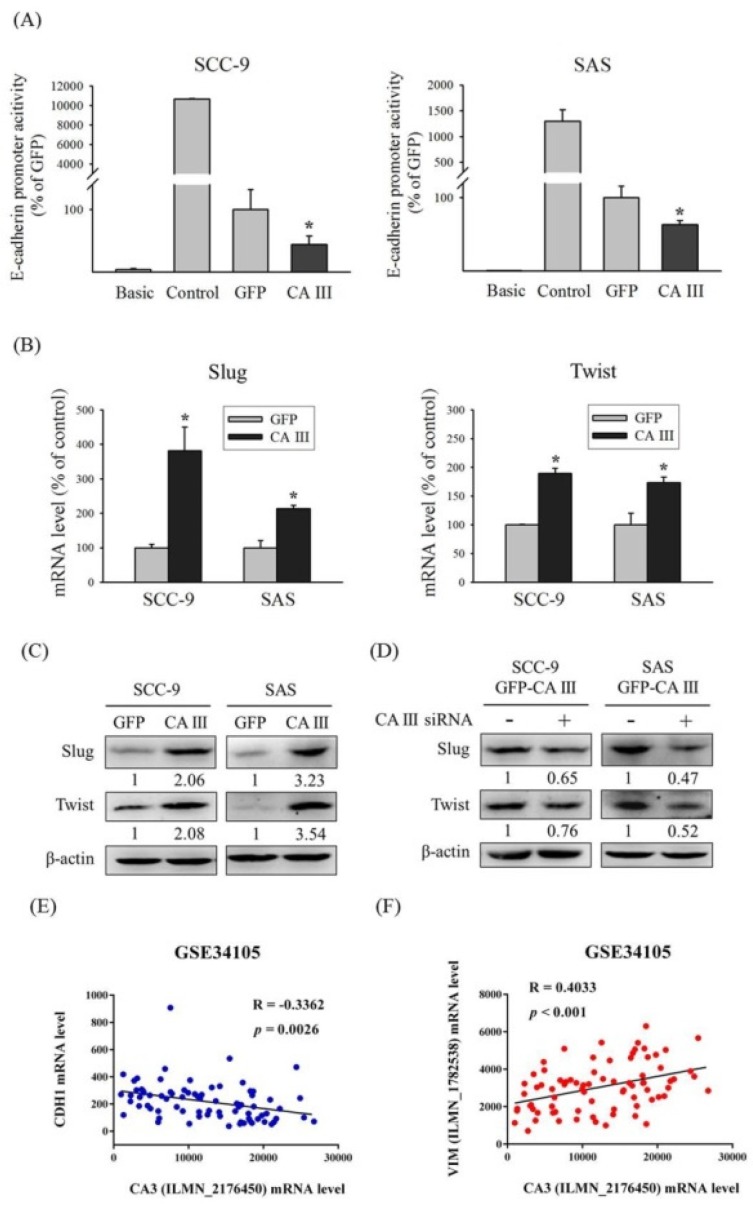
CA III inhibits the promoter activity of E-cadherin and promotes EMT-related transcription factors Slug and Twist in oral cancer cells. (**A**) The E-cadherin promoter activity of SCC-9 and SAS CA III stable cells. The values of luciferase activity were normalized by β-galactosidase expression. * *p* < 0.05 compared with the GFP. (**B**) The mRNA levels of EMT-related transcription factors Slug and Twist were analyzed by real-time PCR. The relative mRNA expression was normalized to GAPDH. * *p* < 0.05 compared with the GFP. (**C**) The protein expression of EMT-related transcription factors Slug and Twist was analyzed by Western blot. β-actin was used as the loading control. (**D**) The protein expression of EMT-related transcription factors Slug and Twist after transfection with scrambled siRNA or CA III siRNA in CA III stable cells. β-actin was used as the loading control. (**E**) Correlation between CA III and CDH1 mRNA expression in the oral cancer tissue from the GEO database. (**F**) Correlation between CA III and VIM mRNA expression in the oral cancer tissue from the GEO database.

To further demonstrate that CA III regulates E-cadherin expression by suppressing the transcription activity of the E-cadherin promoter, we used a luciferase assay to observe E-cadherin promoter activity in the CA III overexpression system. The results indicated that E-cadherin promoter activity was decreased in the CA III overexpression group compared with the GFP control group in both oral cancer cell lines (Figure 3A). Transcription factors may regulate gene transcription by binding on the DNA promoter binding sites. Several EMT-related transcription factors, such as Slug and Twist, were considered to play roles in the EMT process. After CA III overexpression, Slug and Twist expression was significantly higher than both the protein and mRNA level in the GFP control group (shown in Figure 3B and C). Moreover, the protein expressions of Slug and Twist were reversed after CA III knockdown (Figure 3D). According to the aforementioned findings, through the effect of the transcription factors Slug and Twist, CA III could block the E-cadherin promoter transcription activity results of the EMT and stimulate oral cancer cell invasion and migration abilities. In addition, we analyzed the correlation between CA III and EMT markers by using the GEO database to confirm our results. Data from the GEO database GSE34105 demonstrated that CA III mRNA is negatively correlated with CDH1 mRNA (Figure 3E), but is positively correlated with VIM mRNA (Figure 3F).

### 3.4. CA III Promotes the Migration Ability Through the FAK/Src Pathway in Oral Cancer Cells

**Figure 4:**
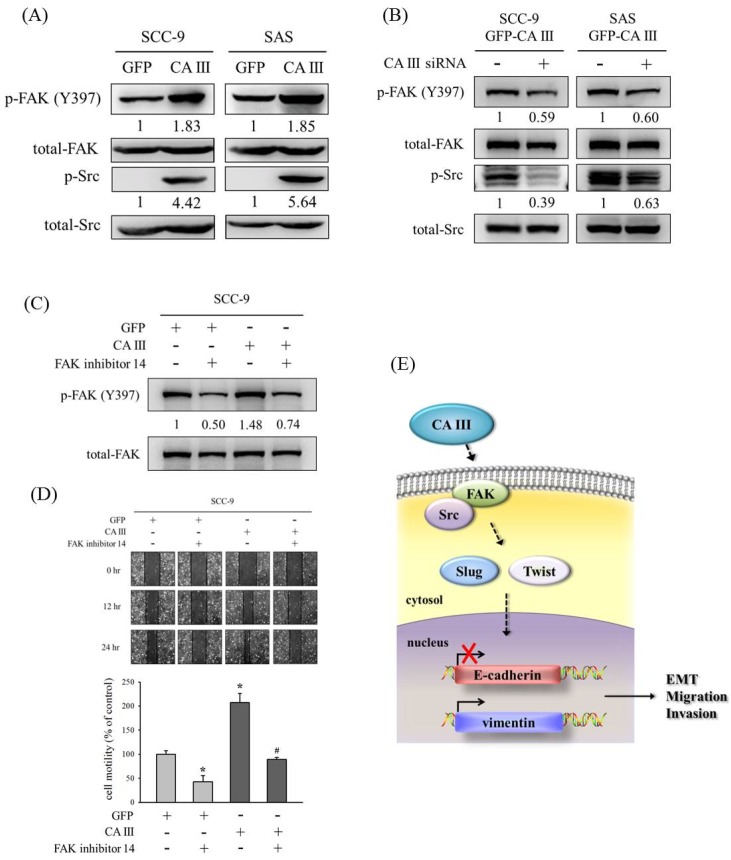
CA III promotes the migration ability via the FAK/Src pathway in oral cancer cells. (**A**) The protein expressions of p-FAK (Y397) and p-Src were analyzed by Western blot. β-actin was used as the loading control. Total-FAK and total-Src were used as the loading control. (**B**) The protein expression of p-FAK (Y397) and p-Src after transfection with scrambled siRNA or CA III siRNA in CA III stable cells. Total-FAK and total-Src were used as the loading control. (**C**) The protein expression of p-FAK (Y397) after treatment of the FAK inhibitor 14 for 24 h. β-actin was used as the loading control. (**D**) SCC9-CA III stable cells after the treatment of FAK inhibitor 14 for 24 h were wounded for 24 h. Phase-contrast pictures of the wounds at three different locations were taken. * *p* < 0.05 compared to GFP stable cells with DMSO. ^#^ *p* < 0.05 compared to CA III stable cells with FAK inhibitor 14. (**E**) Proposed model for how CA III contributes to the EMT, migration, and invasion abilities in oral cancer.

Numerous studies have speculated that the FAK/Src pathway participates in oral cancer migration [34,35,36,37]. Therefore, we also determined whether CA III could regulate the EMT and migration ability though Src and FAK signaling pathways. Results from the Western blot suggested that p-FAK (Y397) and p-Src increased in CA III overexpression cell lines (Figure 4A). In addition, the protein expressions of p-FAK (Y397) and p-Src were decreased after CA III knockdown by CA III siRNA (Figure 4B). Next, we used an FAK inhibitor (FAK inhibitor 14) to confirm whether CA III regulated the migration ability through the FAK pathway. The protein expression of p-FAK (Y397) was increased in CA III overexpression cell lines and decreased after FAK inhibitor treatment (Figure 4 C). Moreover, the cell migration ability also increased in CA III overexpression oral cell lines and decreased after p-FAK (Y397) inhibition (Figure 4 D).

## 4. Discussion

Oral cancer is currently the fourth leading cause of cancer-related deaths in males in Taiwan [38]. The 5-year survival rate for oral cancer is only 50%. Therefore, it is important to identify new prognostic and predictive markers in oral cancer. According to these findings, we suggest that CA III may influence the EMT process by inhibiting the epithelial marker E-cadherin gene transcription binding site affinity, and thus decreasing E-cadherin expression. Moreover, CA III may increase the expression of the mesenchymal markers vimentin and several transcription factors of Slug and Twist through the FAK/Src signaling pathway to stimulate cell invasion and migration abilities similar to tumor metastasis. Relevant studies have suggested that lower CA III expression suppresses cancerous lesions in hepatoma-bearing rats in vivo and that the suppression of CA III accompanies hepatocarcinogenesis [39,40]. However, another study demonstrated that CA III could promote downstream hepatoma cell Sk-Hep1 transformation and invasion abilities through active FAK signaling pathways [29], which is similar to our findings. These different results may be due to the variety of CA III functions in living subjects, such as the regulation of ion transport, the pH value, and water homeostasis to stabilize basic living conditions and function as a tumor suppressor. In vitro, however, CA III may only function as a regulator for cultures and render oral cancer cells more active in invasion and migration.

EMT is known to play critical roles in OSCC carcinogenesis and cancer metastasis [15,41,42,43,44]. Our results showed that the overexpression of CA III protein significantly increased the migration and invasion abilities in oral cancer cells (Figure 1). Moreover, our mRNA array data showed that E-cadherin and vimentin displayed obvious expression differences that were related to EMT (Figure 2 A). In clinical samples of oral squamous cell carcinoma, Chaw et al., observed that decreased E-cadherin expression, but increased vimentin expression, correlated with increased disease severity in OSCC [43]. Costa et al., reported that a reduced expression of E-cadherin was detected at the invasive front and was associated with histological invasiveness in OSCC [44]. Bu et al., also reported that TGF-β1 promotes cell migration by inducing epithelial–mesenchymal transformation in OSCC [45]. However, our mRNA array data showed that TGF-β1 is downregulated on CA III overexpression cells (Figure 2 A). Therefore, the difference in TGF-β1-induced EMT and CAIII-induced EMT in oral cancer needs to be further elucidated in the future.

OSCC metastasis can be regulated though many signaling pathways, such as MAPK, PI3 K/AKT, and FAK/Src [35,36,37,46,47]. Our results showed that the cell migration ability was increased in CA III overexpression cell lines and decreased after FAK inhibitor treatment (Figure 4 C). Consistently, in oral cancer cell lines, Yadav et al., demonstrated that IL-6 could down-regulate E-cadherin expression to promote EMT and metastasis via the FAK signaling pathway [48]. Another study revealed that activated FAK led to increased lymphangiogenesis and lymph node metastasis and promoted EMT in human OSCC cells [49]. Similarly, Xiao et al., also reported that the knockdown of FAK inhibits the invasion and metastasis of oral cancer cell lines by inhibiting the EMT [50]. Additionally, recent advances suggest that FAK-targeting pathways constitute potential anticancer strategies [51,52]. Therefore, the induction of EMT via FAK pathways is a crucial pathway in the tumor metastasis of OSCC.

## 5. Conclusions

In conclusion, our findings suggest that the overexpression of CA III promotes the EMT, migration, and invasion abilities of oral cancer cells through the FAK/Src signaling pathway and transcription factors Slug and Twist, as well as decreases E-cadherin expression and increases vimentin expression (Figure 4 E).
